# A Comparative Study of Benchtop and Portable NIR and Raman Spectroscopic Methods for the Quantitative Determination of Curcuminoids in Turmeric Powder

**DOI:** 10.3390/foods11152187

**Published:** 2022-07-22

**Authors:** Putthiporn Khongkaew, Jordi Cruz, Judit Puig Bertotto, Vanessa Cárdenas, Manel Alcalà, Nantana Nuchtavorn, Chutima Phechkrajang

**Affiliations:** 1 Department of Pharmaceutical Chemistry, Faculty of Pharmacy, Mahidol University, Bangkok 10400, Thailand; putthiporn@go.buu.ac.th (P.K.); nantana.nuc@mahidol.ac.th (N.N.); 2 Faculty of Pharmaceutical Science, Burapha University, Chonburi 20131, Thailand; 3 Salesian School of Sarria, Escola University, 08017 Barcelona, Spain; jcruz@euss.cat; 4 Analytical Chemistry Unit, Department of Chemistry, The Autonomous University of Barcelona, 08193 Barcelona, Spain; judit.bertotto@gmail.com (J.P.B.); manel.alcala@uab.cat (M.A.); 5 National Institute of Metrology, Bogotá 111321, Colombia; vanessa.cardenase@gmail.com

**Keywords:** curcuminoids, turmeric, food quality, nondestructive analysis, portable NIR, portable Raman

## Abstract

Turmeric consumption is continually increasing worldwide. Curcuminoids are major active constituents in turmeric and are associated with numerous health benefits. A combination of spectroscopic methods and chemometrics shows the suitability of turmeric for food quality control due to advantages such as speed, versatility, portability, and no need for sample preparation. Five calibration models to quantify curcuminoids in turmeric were proposed using benchtop and portable devices. The most remarkable results showed that Raman and NIR calibration models present an excellent performance reporting RMSEP of 0.44% *w*/*w* and 0.41% *w*/*w*, respectively. In addition, the five proposed methods (FT-IR, Raman, and NIR) were compared in terms of precision and accuracy. The results showed that benchtop and portable methods were in good agreement and that there are no significant differences between them. This study aims to foster the use of portable devices for food quality control in situ by demonstrating their suitability for the purpose.

## 1. Introduction

**Figure 1:**
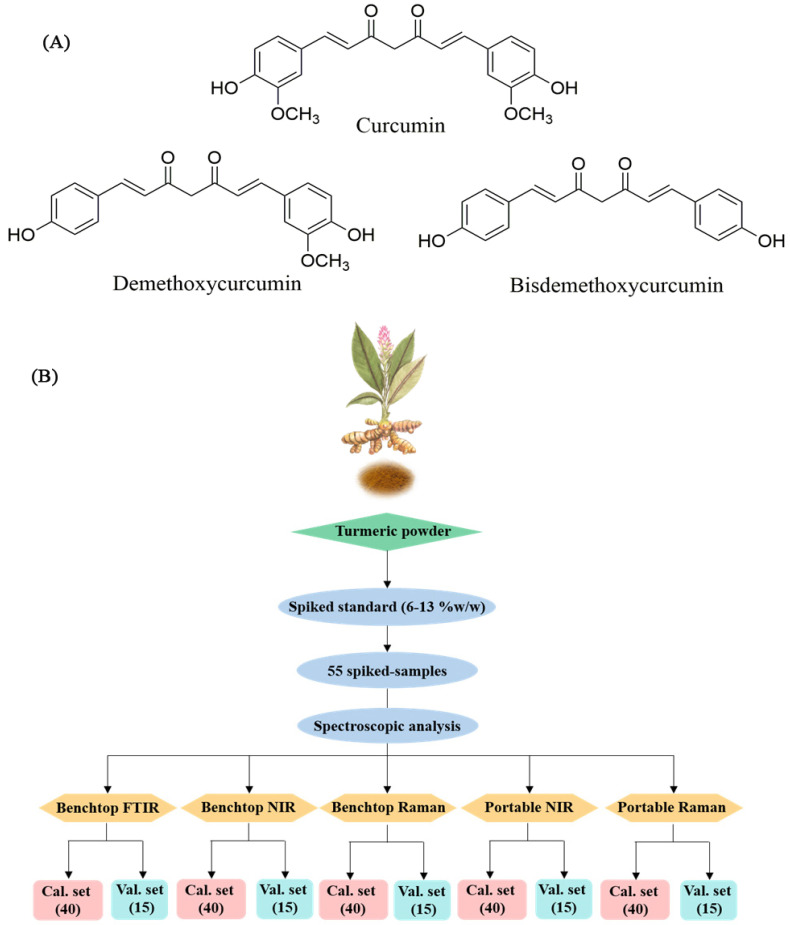
(**A**) Structures of curcumin, demethoxycurcumin, and bisdemethoxy−curcuminand (**B**) diagram of spiked−samples preparation.

Turmeric is obtained from the rhizome of *Curcuma longa* L., which is in the Zingiberaceae family [1]. It has a long history of use in India’s and China’s ancient traditional medicines [2,3], and turmeric consumption is continually increasing worldwide. The active turmeric compounds are curcuminoids, including curcumin, demethoxycurcumin, and bisdemethoxycurcumin (Figure 1A). Turmeric provides a characteristic flavor and yellow color, making it useful as an additive in Asian spices and cuisines. Since turmeric has a surprisingly wide range of beneficial properties, there is a great demand worldwide. The adulteration of turmeric products with potentially harmful ingredients or low-quality turmeric raw material has been encountered. For instance, a lead chromate–based yellow pigment has been added to turmeric to enhance the color of a low-quality dried turmeric root. Quality attributes are required for monitoring turmeric powder material to ensure high curcuminoid content without adulteration. Therefore, developing rapid, simple, and effective analytical techniques is necessary to establish an opportune quality control of the material. Several analytical techniques have been reported for the determination of curcuminoids and adulterated substances in turmeric, such as high-performance thin- layer chromatography (HPTLC) [3], high-performance liquid chromatography (HPLC)-UV detection [4,5,6], liquid chromatography–mass spectrometry (LC-MS), UV-Vis spectrophotometry [7,8,9,10], Fourier transform-infrared (FT-IR) spectroscopy [7,11,12], FT-near infrared spectroscopy (FT-NIR) [13,14], FT-Raman spectroscopy [11,12], and nuclear magnetic resonance spectroscopy [7,15]. However, these analytical techniques present several limitations, including sample and reagent consumption, sample destruction, sample preparation requirement, and portability difficulties for in-field analysis.

FT-IR, NIR, and Raman spectroscopies can be used as nondestructive methods for qualitative and quantitative analysis combined with chemometrics. Although these techniques are complementary methods, providing different molecular information, each method can be independently used for the quantitative determination of curcuminoids. Interpretation of FT-IR spectra between 400–4000 cm^−1^ (2500–25,000 nm) is used to examine the presence of functional groups, and chemical bonding, specifically asymmetric vibrations, provides the most intense bands. The NIR spectroscopy is based on the overtones and combination bands produced in the mid-infrared region (4000–12,821 cm^−^^1^, 780–2500 nm). In this area, vibrations of -CH, -OH, -SH, and -NH bonds are observed. Raman spectroscopy is a complementary technique to FT-IR spectroscopy, which performs in the same wavelength region and provides information on weak or IR-inactive functional groups. The symmetric vibrations illustrate the greatest scattering signal.

Currently, portable spectroscopic instruments are commercially available. Although the cost-effectiveness and user-friendliness properties of portable instruments are attractive, their sensitivity and measurement range are not comparable to the benchtop settings [16,17,18]. In addition, portable instruments have enabled for in-field analysis, which can overcome the limitations of benchtop instruments. Therefore, the assessment of the capability of the portable spectroscopic platforms and their performance for intended use are required for nondestructive quantitative analysis.

The main objective of this work was to demonstrate the competency of portable spectrometers, Raman and NIR, for nondestructive analysis of active substances in spices using turmeric powder as the model material. The results were also compared with benchtop platforms. Meanwhile, nondestructive analysis of curcuminoids by modern spectroscopic instruments has been demonstrated, but only a few studies using portable NIR instruments were reported [19]. To the best of our knowledge, there has been one report for portable Raman spectroscopic analysis of curcuminoids [20].

In this work, five partial least regression (PLSR) models for the quantitative determination of total curcuminoids in turmeric powder were developed using three benchtop instruments (FT-IR, Raman, and NIR) and two portable instruments (Raman and NIR) with the reference values from HPLC method. The main novelty of our study is, to the best of our knowledge, that this is the first comparative study between benchtop and portable spectroscopic instruments for the quantitative determination of total curcuminoids in turmeric powder.

## 2. Materials and Methods

### 2.1. Sample Preparation

Fresh roots of turmeric (*Curcuma longa* L.) were collected from the Wang Nam Yen district, Sakaeo province, Thailand, in January 2018. The roots were washed and dried at room temperature. After that, the dried roots were ground using an electric mill and passed through a sieve with a mesh number of 20, which approximately represents a particle size of 850 µm. This sample was identified by comparing the thin-layer chromatographic (TLC) fingerprint of the authentic sample according to Thai herbal pharmacopeia 2019. A silica gel GF254 was used as the stationary phase, and the mobile phase was the mixture of benzene: chloroform: ethanol (49:49:2% *v*/*v*/*v*). The sample powder was also evaluated with an electron microscope to confirm the characteristics of *Zingiberaceae* roots. HPLC analysis was carried out to determine the total curcuminoid content of turmeric powder, and this lot of turmeric powder was used throughout this study.

The curcuminoid standard (Merck, Germany) and turmeric powder were separately ground and sieved. The basal content of the total curcuminoids in turmeric powder was determined using the HPLC method. Next, an accurate amount of each was mixed by geometric dilution to obtain final concentrations of 6–13% *w*/*w* of curcuminoids in turmeric powder. A total number of 55 spiked samples were prepared in this manner.

Fifteen from 55 spiked samples were randomly selected and used as fixed validation samples to compare all the method performances. The remaining 40 spiked samples were used as calibration samples (Figure 1B).

### 2.2. HPLC Analysis

A high-performance liquid chromatograph (Shimadzu Corporation, Kyoto, Japan) consists of an LC-20AD pump, a SIL-20A autosampler, a CTO-20A column oven, and a SPD-M20A photodiode array detector. The HPLC conditions for the determination of curcuminoids in turmeric powder were modified from the existing publication [21]. The final conditions consisted of a C18 column (4.6 × 150 mm, 5 µm) and a mobile phase mixture of 1% acetic acid and acetonitrile (55:45, *v*/*v*). Isocratic elution was employed with a 1.2 mL/min flow rate and UV detection wavelength at 425 nm. The injection volume was 20 µL.

According to Eurachem guidelines, the method was validated in terms of specificity, linearity, accuracy, and precision. A calibration curve of curcuminoids was established in the range of 10.0–100.0 µg/mL. Accuracy and precision were evaluated by the standard addition method at three different concentrations. The spiked samples were extracted with 25 mL methanol and centrifuged at 5000 rpm for 10 min. Then a 2 mL supernatant was diluted with 50% methanol to 25 mL before HPLC analysis.

Repeatability and intermediate precisions were evaluated at the same concentration level on three different days. Then the validated HPLC method was used as the reference method.

### 2.3. Benchtop NIR Spectroscopy

The spectra were recorded using a Model 5000 spectrometer from FOSS NIR System (Silver Spring, MD, USA) equipped with Rapid Content Analyzer. A glass cuvette for reflectance measurement was filled with 3 g of sample and slightly pressed, leveling the sample powder in the glass. The absorbance was recorded from 1100 to 2498 nm every 2 nm. Nine replications were performed, and the average spectrum of each sample was further used.

### 2.4. Benchtop FT-IR Spectroscopy

The IR spectra of all samples were directly measured by a Nicolet iS5 FT-IR spectrophotometry (Thermo Scientific, Waltham, MA, USA) with attenuated total reflectance (ATR) mode. Twenty mg of the sample was added to the diamond ATR crystal, and the spectrum was collected using deuterated triglycine sulfate (DTGS)detector. The optical speed was 0.482 cm^−1^, and the gain setting was 1. The percent of transmittance was recorded from wavenumber 400 to 4000 cm^−1^ (2500 to 25,000 nm). The diameter of the measurement area was 1.8 mm.

### 2.5. Benchtop Raman Spectroscopy

The spectra were recorded with the Raman spectrometer (Horiba Scientific instrument, LabRAM HR Evolution, Kyoto, Japan). The samples were compressed using a hydraulic press with a force of 10,000 pounds for 30 s to form a circular disc. A 785 nm (red) laser with a deviation angle (DV) and a diffraction grating of 500 nm was used. The Raman spectra were collected from Raman shift 50 to 1800 cm^−1^ (788 to 914 nm) using a 10× objective lens. The laser power was 10%, and the confocal hole was 1000.03. Each Raman spectrum was acquired using an integration time of 10 s and a time average (accumulations) of 3. The diameter of the measurement area was 3.8 µm, calculated according to Equation (1).

Laser spot diameter = 1.22 λ/NA(1)

where λ is the wavelength of the laser and NA is the numerical aperture of the microscope objective being used.

### 2.6. Portable NIR Spectroscopy

A portable NIR spectrometer (MicroNIR, ViaviSolutions^®^, Scottsdale, AZ, USA) with MicroNIR Pro 1700 software was used for the spectral measurement. The glass cuvette for reflectance measurement was filled with 3 g of sample powder. The sample was lightly pressed; thus, it was evenly filled up to the glass. Absorbance values were recorded every 6 nm from 908 to 1676 nm. Similar to the benchtop NIR spectroscopy, three replicates were taken from each sample, and each replication was recorded 3 times. Then, the 9 spectra were averaged and used to represent a unique spectrum from each sample.

### 2.7. Portable Raman Spectroscopy

The Raman spectra were acquired using a portable Raman spectrometer (i-Raman^®^ Plus, B&W TEK, Newark, DE, USA) with a laser of 785 nm. The three controlled parameters included the integration time (acquisition time 4000 ms), the average time (accumulation 5 times), and the laser power (40%, approximately 135 mW). The spectra were collected from Raman shift 0 to 3340 cm^−1^ (785 to 1064 nm). For each sample, three aliquots of approximately 3 g of turmeric powder were taken, and a triple measurement of Raman spectra was performed for each aliquot.

### 2.8. PLSR Model

PLSR models were performed with Unscrambler^®^ program (Aspen Tech, Bedford, MA, USA). The concentration of total curcuminoids in the sample was obtained from the HPLC analysis and used as reference values for the model. Next, 15 from 55 spiked samples were randomly selected and used as fixed validation samples to compare all the method performances. The remaining 40 spiked samples were used as calibration samples. Outliers in the calibration set were detected by using the function F-Residuals and Hotelling’s T2 in the Unscrambler program. In this manner, the remaining samples in the calibration set are 39, 37, and 38 for benchtop NIR, benchtop FT-IR, and benchtop Raman, respectively. In addition, the Hotelling’s T2 statistic at 5% significant level was also used to judge outliers in the prediction set.

The spectral pretreatments used to construct the models included standard normal variate (SNV), Savitzky-Golay Derivatives with second-order polynomial fitting (2D), smoothing (moving average, MA), orthogonal signal correction (OSC), and linear baseline correction (BLC). Consequently, the pretreated spectra were evaluated using principal component analysis (PCA). The main objective of the exploratory analysis was to find the combination of pretreatments that provided better discrimination between concentration ranges.

Calibration models were constructed using the partial least squares regression (PLSR) algorithm and internally validated by cross-validation (the leave-one-out method). The prediction capacity of the models was evaluated by using the validation set samples by the relative standard error of prediction (RSEP) and the root mean squared error of prediction (RMSEP) (Equations (2) and (3)). In addition, bias (Equation (4)) was evaluated to obtain a better perspective of the model performance. Furthermore, the reference and prediction values were statistically analyzed by the *t*-test at a significance level of 0.05.

(2) $$\mathrm{RSEP}(\%)=\sqrt{\frac{\sum_{i=0}^{n}{\left(y_{i}-{\hat{y}}_{i}\right)}^{2}}{\sum_{i=0}^{n}{\left(y_{i}\right)}^{2}}}\times 100$$

(3) $$\mathrm{RMSEP}=\sqrt{\frac{\sum_{i=0}^{n}{\left(y_{i}-{\hat{y}}_{i}\right)}^{2}}{n}}$$

(4) $$\mathrm{Bias}=\frac{\left(y_{\mathrm{ref}}-y_{\mathrm{pred}}\right)}{y_{\mathrm{ref}}}$$

where y_i_ is the reference value for validation set sample i, ŷ_i_ is the predicted value for validation set sample i, and n is the number of samples in the validation set.

The optimum number of factors was determined from a plot of the explained variance against the number of factors. Then the initial model was refined by selecting those factors resulting in the lowest relative standard error for prediction (RSEP), RMSEP, and bias for the validation set.

## 3. Results and Discussion

Since this study’s main objective was to evaluate the competency of portable spectrometers and compare them with benchtop platforms, the samples from one source were used throughout the study to avoid deviation from different sources of samples.

### 3.1. HPLC Analysis

The HPLC method was used as the reference method for the determination of curcuminoid content in turmeric samples. By using the optimum condition, bisdesmethoxycurcumin, desmethoxycurcumin, and curcumin were completely separated with the retention time of 6.26, 6.94, and 7.70 min, respectively. The method was validated according to Eurachem guidelines, and all validation parameters were accepted for the intended purpose (Table 1). Then the collected samples were assessed, showing a curcuminoid content of 6% *w*/*w*. These samples were used throughout the study.

### 3.2. Benchtop vs. Portable Spectroscopic Platforms

In this study, three spectroscopic methods based on benchtop platforms (i.e., FT-IR, NIR, and Raman spectrometers) and two methods based on portable platforms (i.e., NIR and Raman spectrometers) were developed for the quantitative determination of curcuminoids in turmeric powder. The suitability of the different instruments and the accuracy between benchtop and portable platforms for the same instrument were compared. Compared with NIR and FT-IR spectroscopy, Raman spectroscopy has limitations due to the colored sample’s fluorescence properties. This fluorescence can seriously interfere with the detection of scattered Raman photons, making it difficult to obtain information from the spectra.

Hence, optimizing the laser power for the Raman spectra acquisition was necessary; this did not irradiate the turmeric samples possessing yellow/orange color with relatively strong laser light.

### 3.3. Benchtop Platforms

**Figure 2:**
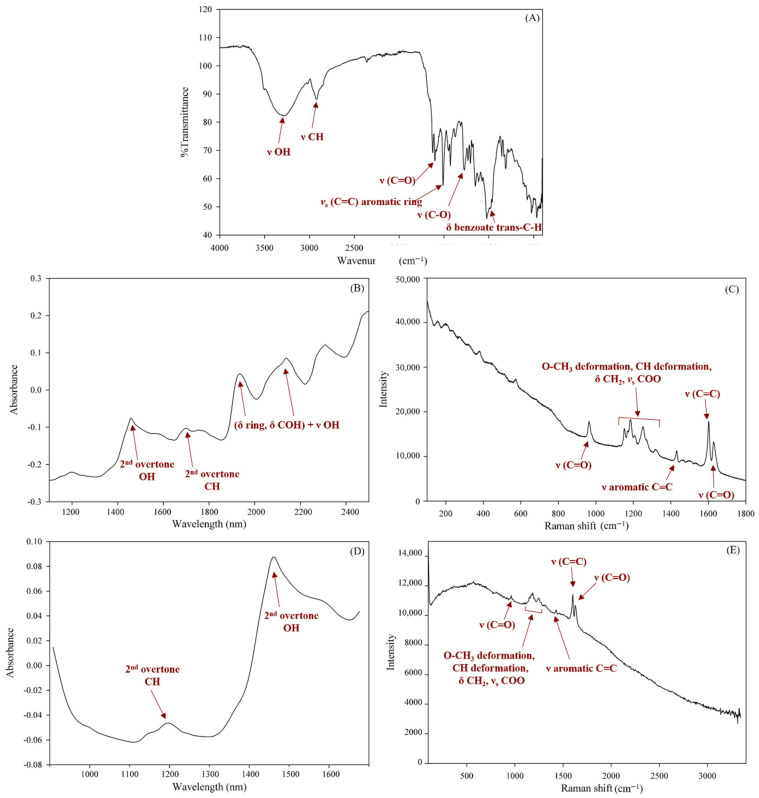
Spectra obtained from (**A**) benchtop FT-IR, (**B**) benchtop NIR, (**C**) benchtop Raman, (**D**) portable NIR, and (**E**) portable Raman spectrometer.

For the comparison of the spectra profiles obtained from the three benchtop instruments, the raw spectral data of the same sample with the characteristic bands of the curcuminoids are presented in Figure 2 [20,22,23]. For Raman spectra (Figure 2C,E), the characteristic bands agreed with the existing publication [20].

The final Figure of merit employed for the spectroscopic method and the PLS models is shown in Table 2. All the models presented a low number of PLS factors (excepting benchtop Raman spectroscopy), low RMSEC, and good *R*^2^. In addition, the calibration regression line was statistically tested with the slope equal to 1 and the intercept to zero. Furthermore, all the models were statistically validated. The calibration and prediction factor plots of the optimum models are presented in Figure S1 of the Supplementary Data. The prediction results, the limit of detection (LOD), and the limit of quantitation (LOQ) of the optimum models are shown in Table S1. Table 2 also shows the statistical attributes used to compare the performance of all the instruments. The benchtop NIR and FT-IR spectroscopic methods represented a good predictive capability. Moreover, all the benchtop models provided good RSEP prediction errors between 4 to 6%, and the bias approached, demonstrating no systematic prediction error.

Additionally, the t-test of residuals was performed, and all the t-experimental values were lower than the t-critical values, showing no significant differences between predicted values and HPLC reference values (Table 3).

**Figure 3:**
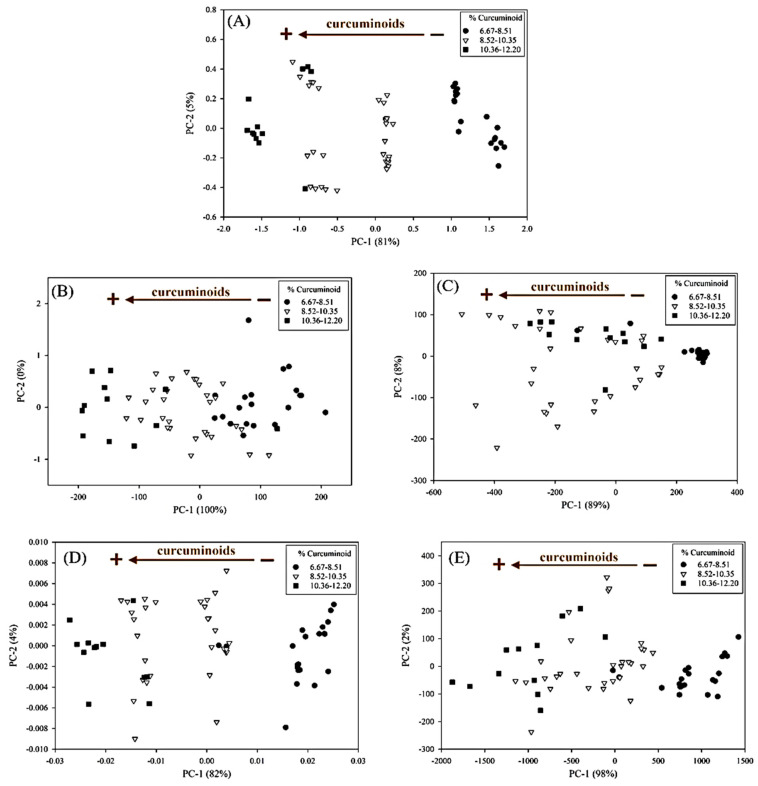
PCA of the pre-processed data using (**A**) SNV+2D of benchtop NIR spectral range of 1854–2260 nm, (**B**) OSC with 2 factors of benchtop FT-IR spectral range of 766–1687 cm^−1^, (**C**) smoothing + BLC + OSC with 1 factor of benchtop Raman spectral range 840–1799 cm^−1^, (**D**) SNV + 2D of portable NIR spectral range of 970–1645 nm, and (**E**) MA + BLC of portable Raman spectral range of 800–2013 cm^−1^.

**Figure 4:**
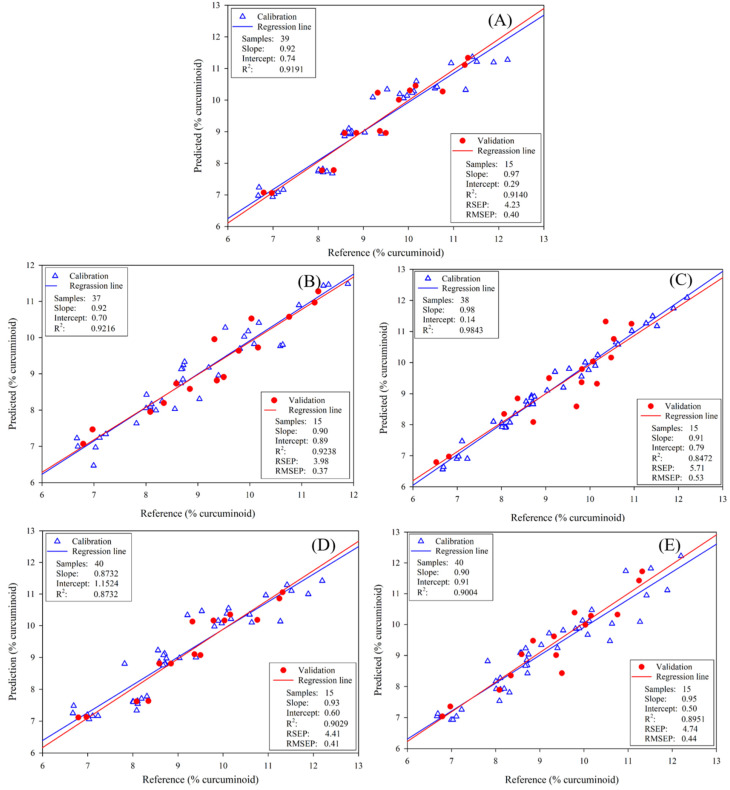
Calibration and validation curves of predicted curcuminoid values vs. reference obtained from the PLS model after the pre-processed data as described in Figure 3.

For FT-IR spectroscopy, the optimum PLSR model was obtained by using a spectral range of 766–1687 cm^−1^ (5928–13,055 nm) with OSC data pretreatment (Figure 3B). The OSC is a pre-processing method that can remove variation from the X-data, which is unrelated to the interest response [24]. For FT-IR data, OSC can remove systematic variation from the response matrix (%T) that is unrelated or orthogonal to the property matrix (concentration). This pretreatment method offered a good PLSR model with 1 PLS factor (Figure 4), the RSEP value of 3.96%, the RMSEP of 0.373, and the bias of 0.046. Compared with the NIR spectroscopic model, the prediction power obtained from the FT-IR spectroscopic model was slightly superior to the NIR spectroscopy, whereas there was no statistically significant difference between these two models.

However, the benchtop Raman spectroscopic model (Table 2) required a higher number of PLS factors than the NIR and FT-IR spectroscopic models. Figure 3C presents the PCA plot of all samples acquired with the benchtop Raman spectroscopy. There was no correlation regarding turmeric content, since all concentrations were mixed in the score plot; hence, the samples were not discriminated for their concentration as happened with other spectroscopic techniques in this study.

This was due to the small spot diameter of the measurement probe or radiation spot (3.8 µm) of the benchtop Raman spectroscopy, which was smaller than the other methods in this study. Thus, the small spot area might not be representative of the sample. Also, the heterogeneity of the turmeric samples was crucial for the Raman spectroscopic measurements.

### 3.4. Portable Spectroscopic Platforms

The final figure of merit for the PLS models of the portable instruments and the statistical attributes used to compare the performance of all instruments is presented in Table 2. All the models show a low number of PLS factors, low RMSEP, and good *R*^2^. Furthermore, all the models were statistically validated.

Both portable Raman and NIR spectroscopic models provided good RSEP prediction errors between 4 and 5%; the bias approached 0, representing no systematic prediction error. Moreover, a *t*-test of the residuals was performed, and all the *t*-experimental values were lower than the *t*-critical values, showing no significant differences between predicted values and HPLC reference values. Thus, the models of portable platforms could predict the test set similarly to the benchtop platforms.

After comparing the statistical attributes of the prediction of all portable models, we concluded that the best model was obtained from NIR spectra with a spectral range of 970–1645 nm using SNV + 2D Savitzky-Golay derivative with 7 points window as the optimal pretreatment combination. The model was calculated with 1 PLS factor (Figure 4), and it performed a RSEP value of 4.41%.

### 3.5. Comparison of Benchtop and Portable Spectroscopic Platforms

The proposed analytical methods were compared in terms of precision and accuracy using the ValidaR application [25,26,27]. A comparison was also performed between the variances of the prediction results obtained from the benchtop and the portable NIR and Raman spectroscopic methods. The analysis of variance (ANOVA) showed no significant differences between the models (Table 3).

The results of the F-test showed no differences between the variances. Therefore, it can be presumed that there were no differences in precision between the portable and benchtop platforms. Furthermore, regarding the method accuracy, the comparison of means between benchtop and portable platforms of both NIR and Raman spectroscopy was carried out; there were no differences between those platforms.

In terms of model parameters, the higher *R*^2^ of the model and lower RMSEC indicated the good fit of model for the new samples. In addition, a higher *R*^2^ Pearson and lower RMSEP and RSEP values indicated good correlation between predicted and reference values. However, a higher *R*^2^ model does not guarantee a higher *R*^2^ Pearson, as shown from the benchtop Raman model.

Among all the investigated methods, for the benchtop setting, FT-IR and NIR were superior to Raman for the quantitative determination of total curcuminoids in validation samples. This might be due to the small spot diameter of the measurement probe of Raman and the color sample’s fluorescence problem. For portable instruments, the portable NIR spectrometer’s performance was slightly better than the portable Raman for the quantitative analysis of curcuminoids in turmeric samples. The predictive capability for portable instruments was not significantly different from the best PLSR model obtained from the benchtop FT-IR spectrometer.

When the same spectroscopy types are compared, the results in Table 1 show that the models of benchtop NIR and Raman were inferior to portable NIR and Raman. The model parameters and method performance values were slightly better for benchtop compared to portable instruments.

## 4. Conclusions

In past decades, complicated analysis methods in several branches have been allowed with modern nondestructive analytical methods [28,29]. In this study, five different spectroscopic methods, including benchtop FT-IR, NIR, and Raman spectrometers and portable NIR and Raman spectrometers combined with PLSR, were investigated to analyze the total curcuminoid content in turmeric samples. The benchtop instruments, FT-IR, and NIR spectroscopy–based PLSR models illustrated the best predictive capabilities. Portable NIR and Raman spectroscopies showed no statistically significant differences between benchtop instruments; the same result obtained with the HPLC method. The proposed portable spectroscopy methods combined with PLSR models were successfully applied to the quantitative determination of curcuminoids in turmeric powder. Moreover, the methods performed by portable instruments offered low cost, rapidity, and nondestructive analysis, and could potentially be used for in-field analysis without any sample preparation steps for the quality control of the turmeric. Our findings clearly confirmed that each spectroscopic instrument combined with the chemometric approach was able to quantify total curcuminoid content, and portable instruments more efficiently obtained the quantitative determination data compared with benchtop instruments.

## Figures and Tables

**Table 1:** The validation results of the HPLC method.

Specificity			
Name	Retention Time (min)	Resolution *	Peak Purity (%) *
Standard			
bisdemethoxycurcumin	6.26	-	98.7
demethoxycurcumin	6.94	2.4	99.0
curcumin	7.70	2.5	98.9
Turmeric sample			
bisdemethoxycurcumin	6.27	-	96.0
demethoxycurcumin	6.95	2.5	97.8
curcumin	7.70	2.4	97.1
*t*-test (*p*-value > 0.05)	0.99		
Linearity			
Linear equation	y = 130,514x − 640,682		
Correlation coefficient (*r*) **	0.9999		
Accuracy (%recovery ± SD) ***			
Curcuminoid spiked concentrations	Repeatability (*n* = 3)	Intermediate precision (*n* = 3)	
5 μg/mL	101.0 ± 0.7	100.7 ± 0.6	
35 μg/mL	101.5 ± 1.3	100.0 ± 1.6	
74 μg/mL	100.2 ± 0.2	99.2 ± 1.0	
Precision (%RSD) ****	1.0	0.8	

* Resolution and peak purity should be ≥2.0 and 95.0%, respectively; ** Correlation coefficient (*r*) should be ≥0.999; *** %Recovery should be within 95–102%; **** %RSD should be less than 2.0%.

**Table 2:** PLSR models and performance of all the methods.

Model Parameters	Spectroscopic Method				
	Benchtop NIR	Benchtop FT-IR	Benchtop Raman	Portable NIR	Portable Raman
Spectral region	1854–2260 nm	766–1687 cm^−^^1^	840–1799 cm^−^^1^	970–1645 nm	1536–1696 cm^−^^1^
Pre-treatment	SNV + 2D(11p)	OSC 2 factors	Smoothing + BLC + OSC 1 factor	SNV + 2D(7p)	MA(5p) +BLC
PLS factors	1	1	3	1	3
Explained variance (%)	92	92	98	87	90
Calibration samples	39	37	38	40	40
Intercept	0.74	0.70	0.14	1.15	0.91
Slope	0.92	0.92	0.98	0.87	0.90
*R*^2^ model	0.9191	0.9216	0.9843	0.8700	0.9004
RMSEC	0.4143	0.3792	0.1851	0.5175	0.4587
Diameter of the measurement area	N/A	1.8 mm	3.8 µm	30 mm	85 µm
**Method performance**					
*R*^2^ (pearson)	0.9140	0.9238	0.8472	0.9029	0.8951
RSEP (%)	4.227	3.983	5.713	4.404	4.739
RMSEP	0.396	0.373	0.535	0.413	0.444
Bias	−0.011	0.046	−0.020	0.063	−0.081
*t*_crit_	2.14	2.14	2.14	2.14	2.14
*t*_exp_	0.009	0.041	0.017	0.054	0.067

**Table 3:** Statistical comparison between methods.

Statistical Evaluation	Spectroscopic Method	
ANOVA	All methods-FT-IR, NIR, Raman	
	F-experimental	F-critic
	0.012	2.5
Comparison of variances (F-Test)	Raman benchtop and micro Raman	
	F-experimental	F-critic
	0.391	2.98
	NIR Benchtop and micro NIR	
	F-experimental	F-critic
	1.079	2.98
Comparison of means (*t*-Test)	Raman benchtop and micro Raman	
	*t*-experimental	*t*-critic
	0.103	2.14
	NIR benchtop and micro NIR	
	*t*-experimental	*t*-critic
	0.173	2.14

## Supplementary Materials

The following are available online at https://www.mdpi.com/article/10.3390/foods11152187/s1, Table S1: Prediction results, LOD and LOQ of the optimum PLSR models and Figure S1: The calibration and prediction factor plots of the optimum PLSR models.
