# Supplementary material for: A Comparative Study of Benchtop and Portable NIR and Raman Spectroscopic Methods for the Quantitative Determination of Curcuminoids in Turmeric Powder
Source: Foods. 2022 Jul 22;11(15):2187. doi: 10.3390/foods11152187 (PMC9331271; doi:10.3390/foods11152187)
Supplement: Supplementary file 1 [file foods-11-02187-s001.zip › foods-1816277-supplementary.pdf]

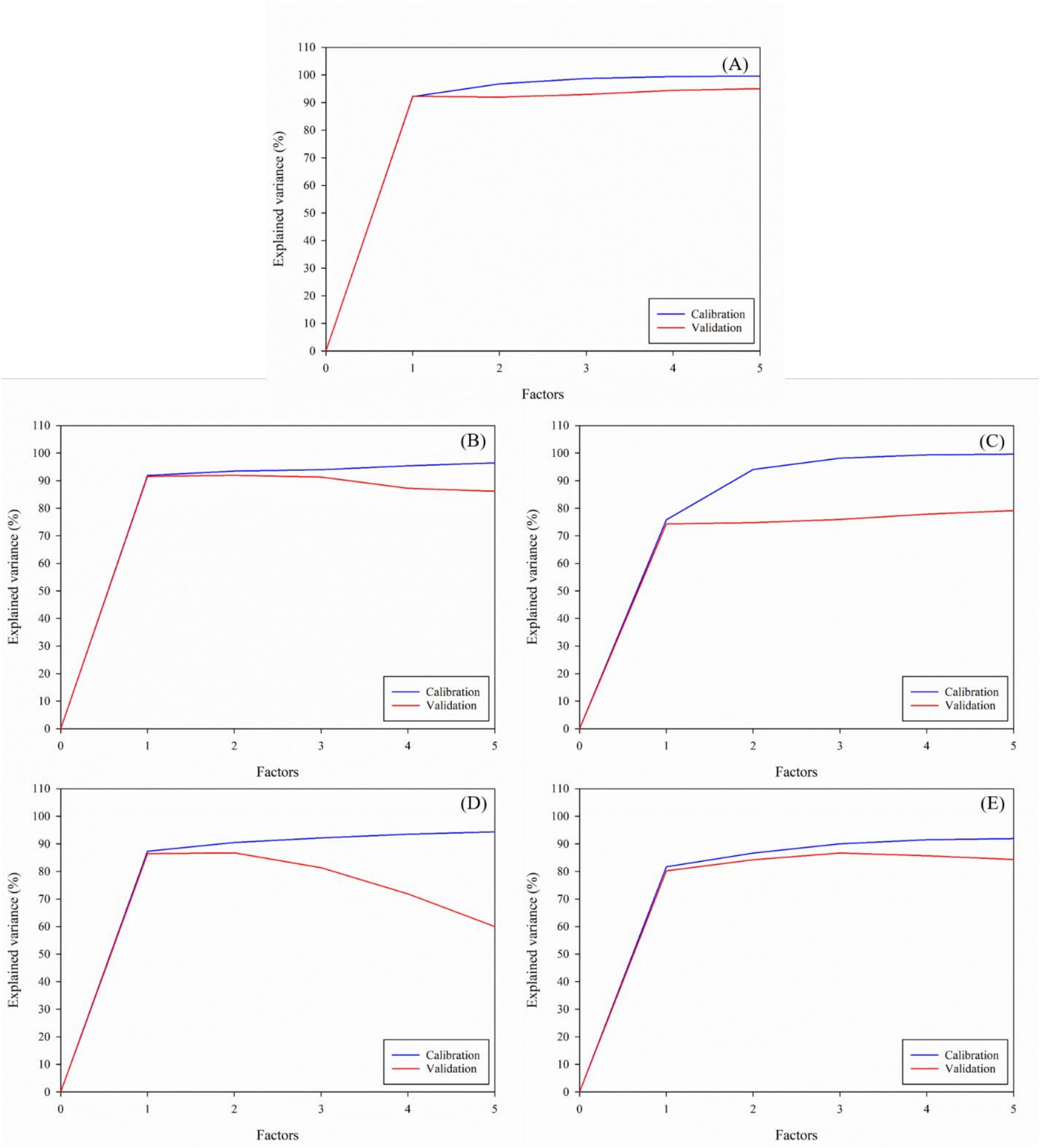

Figure S1. The calibration and prediction factor plots of the optimum PLSR models.

Table S1. Prediction results, LOD and LOQ of the optimum PLSR models

Validation samples	Total curcuminoids concentration (% *w*/*w*)					
	Benchtop NIR	Benchtop FT-IR	Benchtop Raman	Portable NIR	Portable Raman	HPLC (Average ± SD, *n*=3)
T1	7.056	7.467	6.526	7.130	7.355	6.972 ± 0.007
T2	7.071	7.069	6.813	7.115	7.037	6.793 ± 0.002
T3	9.021	8.821	8.718	9.098	9.011	9.374 ± 0.004
T4	8.957	8.913	8.061	9.071	8.428	9.496 ± 0.001
T5	10.008	9.639	9.695	10.159	10.388	9.787 ± 0.002
T6	7.780	8.197	8.363	7.632	8.353	8.341 ± 0.004
T7	7.773	7.953	10.159	7.620	7.889	8.084 ± 0.003
T8	8.956	8.737	9.812	8.810	9.037	8.587 ± 0.003
T9	8.959	8.587	9.075	8.807	9.475	8.845 ± 0.006
T10	10.450	9.726	9.820	10.350	10.281	10.163 ± 0.006
T11	10.231	9.955	10.066	10.128	9.616	9.315 ± 0.019
T12	10.303	10.527	10.479	10.162	9.989	10.027 ± 0.003
T13	10.269	10.575	10.539	10.180	10.321	10.759 ± 0.010
T14	11.337	11.279	10.939	11.057	11.724	11.316 ± 0.003
T15	11.110	10.970	10.356	10.858	11.427	11.253 ± 0.001
LOD*	1.401	1.358	3.541	1.518	1.609	-
LOQ*	4.377	4.114	10.731	4.600	4.876	-

* LOD = (3.3 * σ)/S and LOQ = (10 * σ)/S, where σ is standard error of the plots between HPLC and PLSR determinations and S is the slope of the plot between HPLC and PLSR determinations
